# *Sarcocystis* spp. in Romanian Slaughtered Cattle: Molecular Characterization and Epidemiological Significance of the Findings

**DOI:** 10.1155/2019/4123154

**Published:** 2019-10-13

**Authors:** Kálmán Imre, Gheorghe Dărăbuș, Emil Tîrziu, Sorin Morariu, Mirela Imre, Judit Plutzer, Marius V. Boldea, Adriana Morar

**Affiliations:** ^1^Department of Animal Production and Veterinary Public Health, Faculty of Veterinary Medicine, Banat's University of Agricultural Sciences and Veterinary Medicine “King Michael I of Romania” Timişoara, Timişoara 300645, Romania; ^2^Department of Parasitology and Parasitic Diseases, Faculty of Veterinary Medicine, Banat's University of Agricultural Sciences and Veterinary Medicine “King Michael I of Romania” Timişoara, Timişoara 300645, Romania; ^3^National Public Health Center, Department of Water Hygiene, Budapest 1097, Hungary; ^4^Department of Soil Science Mathematics and Statistics, Faculty of Agriculture, Banat's University of Agricultural Sciences and Veterinary Medicine “King Michael I of Romania” Timişoara, Timişoara 300645, Romania

## Abstract

Species of the genus *Sarcocystis* are recognized as protozoan parasites infecting a wide range of animals, including humans. This study aimed to provide data on the occurrence, genetic characterization, and epidemiological significance of *Sarcocystis* spp. in cattle destined for human consumption in Romania. A total of 117 heart samples from slaughtered cattle in three southwestern Romanian counties (Dolj, Timiș, and Gorj) were analyzed in order to detect sarcocysts, using fresh examination microscopic techniques. Subsequently, the isolated sarcocysts and/or cyst fragments (5–15 per sample) from each infected animal were molecularly characterized. Overall, 17.9% (21/117) of the tested animals were found to be *Sarcocystis* spp. positive by microscopy. Genetic characterization of *Sarcocystis* spp. isolates, based on sequence analysis of the *18S rRNA* gene, showed the presence of a single species, namely *S. cruzi*. No correlation was found (*p* > 0.05) between *S. cruzi* infection and the origin, age, breed, and gender of cattle, but the grazing farming system was positively associated (*p*=0.031) with the pathogen prevalence and can be considered a risk factor (OR = 3.6) in acquiring infection. To evaluate the possible public health risk, further investigation focused on the processing of other *Sarcocystis*-specific tissue matrices and evidence of human infections is recommended. This is the first study of bovine *Sarcocystis* infection in Romania.

## 1. Introduction

*Sarcocystis* spp. are common protozoan (Apicomplexa: Sarcocystidae) parasites, which, during their life cycle, require both definitive (i.e., carnivores and primates including humans) and intermediate (i.e., herbivores and omnivores) hosts [1, 2].

Results of molecular investigations, based on analysis of *18S rRNA*, *cox1*, and *ITS1* gene sequences, have shown that cattle, as intermediate hosts, can usually harbor tissue cysts (sarcocysts) of five *Sarcocystis* species, including *S. cruzi*, *S. hirsuta*, *S. hominis*, *S. rommeli* (formerly known as *S. sinensis*-like), and *S. heydorni* [3]. Among them, *S. hominis* and *S. heydorni* are human pathogens. Canids act as definitive hosts for *S. cruzi* and felids for *S. hirsuta* and *S. rommeli* [4], while humans or other primates for *S. hominis* and *S. heydorni* [2–4]. Of the three genetic markers, only the *18S rRNA* gene sequences are available in GenBank for all the five *Sarcocystis* species known to be responsible for bovine sarcocystosis.

Naturally, infections occurring in cattle have been occasionally associated with eosinophilic myositis [5, 6], fatal eosinophilic myocarditis [7], and economic losses (e.g., reduced milk production, abortion, or neonatal mortality). The infected intermediate hosts can harbor thin-walled (*S. cruzi* and *S. heydorni*), and/or thick-walled (*S. hominis*, *S. hirsuta*, and *S. rommeli*) muscle sarcocysts, easily differentiable with ultrastructural microscopy [3]. The disease in humans, as a consequence of the ingestion of raw and/or undercooked meat, can occur under two forms. The first is the well known and studied intestinal sarcocystosis caused by zoonotic species (e.g., *S. hominis*, *S. heydorni*, or *S. suihominis*), resulting in symptomatic gastroenteritis, vomiting, nausea, and abdominal pain [3]. The other is the less studied muscular sarcocystosis, occurring when humans become dead-end hosts after the accidental ingestion of sporocysts from nonhuman *Sarcocystis* spp. [8, 9].

Epidemiological surveys of *Sarcocystis* spp. in cattle tissues destined to human consumption, using molecular tools, are still lacking in the majority of European countries, including Romania. Results of the few available studies conducted in Italy [10], Germany [11], and Hungary [2], aiming to assess the risk of zoonotic infections, have revealed the dominance of *S. cruzi*, but in each of them, the well-known human pathogen *S. hominis* has also been reported. According to European Union legislation, the screening for sarcocysts within the meat inspection is not mandatory. This aspect can greatly contribute to the poor understanding of the parasite spreading within the bovine populations at continental level. These information gaps have been underlined by the European Food Safety Authority (EFSA) within a scientific report, encouraging the monitoring of *Sarcocystis* parasite presence in animals and foodstuffs by each state member [12].

Taking these into consideration, the present study aimed to provide data on the occurrence, molecular characterization, and epidemiological significance of *Sarcocystis* spp. from slaughtered cattle in Romania.

## 2. Materials and Methods

The study was conducted in three southwestern (between 46° 06′ N, 20° 15′ E and 43° 45′ N, 26° 00′ E) Romanian counties (Dolj, Timiș, and Gorj), with a temperate-continental climate, from March 2017 to July 2018, covering an area of about 21.713 km^2^, with a total of 65.514 bovine population. A total of 117 whole heart specimens, reported as being the most relevant predilection sites for the assessment of isolation frequency of *Sarcocystis* spp. [13–15], were collected from slaughtered cattle during veterinary postmortem inspections in two regional slaughterhouses of the screened area. Individual animal-related (age, breed, and gender) and epidemiological (origin and farming system) data (see Table 1) were provided by veterinarians. Cattle from grazing and intensive management systems were examined. The grazing system in the screened region includes cattle form backyard and/or small-scale integrated livestock farms (up to 20–30 heads), from rural areas, in which the animals grazing on rangeland during warm season, with daily access of the possible sporocysts contaminated pastures, fodder, and drinking water. Cattle from intensively managed farming systems (more than 100 heads), with implemented biosecurity practices, generally have a lower access to the excretion of definitive hosts compared with grazing cattle. Before subjecting them to laboratory processing, the samples were stored frozen at −20°C.

On examination day, the hearts were thawed at room temperature. In order to detect sarcocysts, ten grams of myocardium (vertex) was processed, using the fresh examination microscopic technique [1].

In brief, the tested samples were minced in a blender, adding 40 ml of peptone buffered solution (PBS), and stirred for 10 min on a magnetic stirrer for a good homogenization. Next, the mixture was poured through a sieve, collected in a 50 ml tube and centrifuged for 5 min, at 600 ×g. Subsequently, the supernatant was withdrawn and the resulted final concentrated pellet was resuspended in 25 ml PBS. Finally, the pellet was transferred into a Petri dish and examined using light microscopy (100x magnification) in order to identify the possible sarcocysts [1]. Several sarcocysts and/or cyst fragments (5–15) from each microscopically positive sample were collected in microcentrifuge tubes (500 *μ*l), containing sterile distilled water (100 *μ*l), and were stored at −20°C until molecular analysis.

DNA was extracted using the Isolate II Genomic DNA kit (Bioline Reagents Limited®, London, UK), according to the manufacturer's recommendations. Molecular detection of *Sarcocystis* spp. was accomplished through genus-specific polymerase chain reaction (PCR) targeting the highly conserved *18S rRNA* gene (∼915 bp). The 2 L forward (5′-GGATAAACCGTGGTAATTCTATG-3′) and the 3H reverse (5′-GGCAAATGCTTTCGCAGTAG-3′) specific primer set and cycling parameters were used as previously reported [16]. The positive control, which consisted of a good-quality genomic DNA extracted from a single and previously GenBank-deposited (KX008292.1) sarcocysts, together with a negative control, represented by sterile deionised water instead of DNA in the PCR mix, was also included in the reactions. Aliquots of amplified PCR products were analyzed on a 2.2% agarose gel stained with Midori Green™ (Nippon Genetics®; Europe Gmbh). Subsequently, species identification was performed through bidirectional sequencing of the purified (Isolate II PCR and Gel Kit, Bioline®) amplicons by the Macrogen Europe® Company (Amsterdam, the Netherlands). Finally, Basic Local Alignment Search Tool (BLAST) analysis was performed, in order to compare the obtained sequences to those available in the GenBank® dataset. Two *S. cruzi* sequences were submitted to GenBank® (accession numbers MH223459 and MH223460).

Subsequently, a phylogenetic tree was constructed using the Neighbor-Joining algorithm implemented in ClustalW, based on evolutionary distances calculated by the Kimura two-parameter model with 1,000 bootstrap sampling (available online: http://clustalw.ddbj.nig.ac.jp/index.php?lang=en).

The statistical analysis of the variation of *Sarcocystis* spp. prevalence in cattle, in relation to the recorded animal and epidemiological data, was performed with Pearson's chi-square (*χ*2) test, using IBM SPSS Statistics for Windows, Version 21.0 (IBM Corp., Armonk, NY, USA). A *p* value ≤ 0.05 was considered significant. In addition, risk factors were evaluated, using the multivariable regression analysis. Each variable was included in the binary logit model as an independent variable, resulting in the calculation of odds ratios (ORs), with 95% confidence intervals (CIs).

## 3. Results

**Figure 1:**
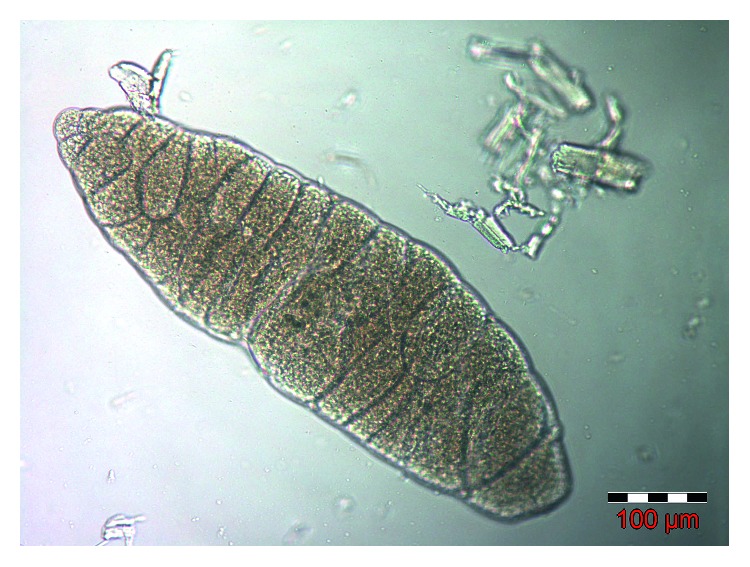
Light microscope appearance (100x magnification) of the thin-walled *S. cruzi* with several internal septa that form compartments containing bradyzoites.

The results of the survey are summarized in Table 1. Overall, a total of 21 out of 117 (17.9%) slaughtered southwestern Romanian cattle were found to be *Sarcocystis* positive, by microscopically fresh examination. The observed elongated ovals (up to 300 *μ*m long and 110 *μ*m wide; [15]) and spherical sarcocysts had a smooth and thin wall, enclosing numerous bradyzoites (Figure 1). These observations were suggestive for *S. cruzi* and/or *S. heydorni* species [3, 15]. *Sarcocystis*-infected animals were recorded in all the screened counties (Dolj: 14.9%; Timiș: 16.7%; Gorj: 22.5%), of all ages (≤2 years: 9.7%; 2 to 8 years: 20%; >8 years: 22.2%) and breeds (Holstein Friesian: 15.6%; Bălțată Românească: 16.7%; crossbreed: 19.7%) (Table 1), but the registered prevalence of the infection did not reach the level of statistical significance (*p* > 0.05). The numbers of infected female and male cattle did not differ significantly from each other (17.7% vs. 20.0%). However, the cattle reared in the grazing system (24.6%, OR = 3.6, 95% CI 1.1—11.5) were found to be more susceptible to *Sarcocystis* infection than those farmed in the intensive system (8.3%, 95% CI 2.7—20.9, *p*=0.031).

**Figure 2:**
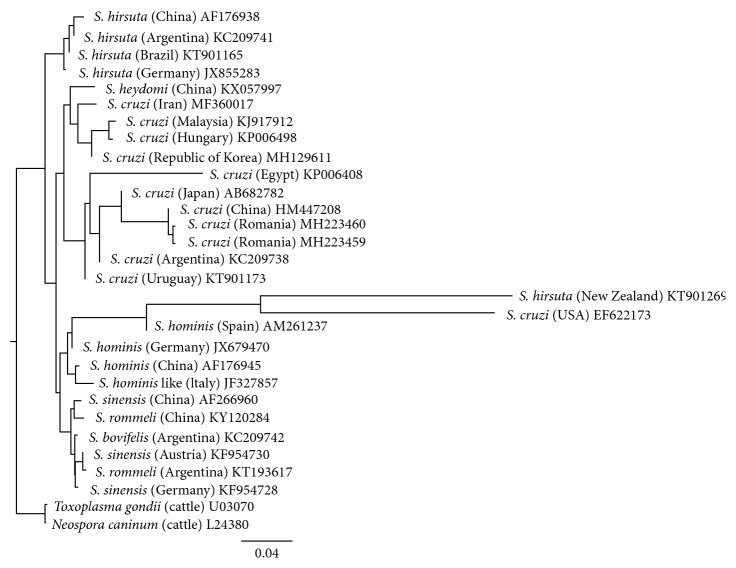
Phylogenetic tree showing the relationship of the *S. cruzi* sequences resulting from the current survey (marked with bold), and other cattle-specific *Sarcocystis* spp. reported in different countries (in brackets), based on analysis of a partial sequence of the *18S rRNA* gene.

Molecular characterization of *Sarcocystis* isolates based on sequence analysis of the *18S rRNA* gene was successfully done in all microscopically positive specimens and indicated that all 21 isolates were positive for *S. cruzi* (with 97–100% homology to GenBank® reference sequences; KT901168.1, JX679467.1, AB682781.1). Analysis of the phylogenetic relationships of our sequences and other GenBank®-retrieved *Sarcocystis* reference sequences recorded in cattle in different countries and/or reported in scientific papers (see Figure 2) showed that our isolates clustered closely with other *S. cruzi* sequences isolated from cattle in Argentina, China, Egypt, Japan, and Uruguay.

## 4. Discussion

To the authors' knowledge, this is the first molecular study of bovine *Sarcocystis* infection in Romania, providing information for the nation and mainland Europe. Contrary to the high overall *Sarcocystis* infection prevalence in cattle registered in other studies (66.2%, [2]; 78.1%, [10]; 100%, [17, 18]; 41.5%, [19]; 97.4%, [20]), in our survey, a considerably lower infection level (17.9%) was recorded. However, it should be taken into account that in some another investigations, a larger amount of tissue samples were processed (e.g., [17, 19, 20]), and the registered cumulative prevalence values are the result of the screening of at least two *Sarcocystis*-specific tissue matrices (e.g., heart, skeletal muscle, esophagus, diaphragm, and tongue [2, 10, 18]), with different diagnosis methods (e.g., light microscopy and fresh, pepsin digestion, and transmission electron microscope examinations), under different combination forms. Likewise, in our study, the direct PCR screening of the final concentrated pellet or heart tissue of the microscopically negative samples, which may increase the detection sensitivity of *Sarcocystis* spp., as highlighted by Pritt et al. [21], would have contributed to the diagnostic precision. Moreover, the pellet screening with a higher power objective lens (e.g., 400x), like in the case in [17], would facilitate the identification of bradyzoites resulted from the ruptured cysts, thus increasing the prevalence of infection. Taking these into consideration, it can be assumed that in our study, the true prevalence is underestimated. Similar to our findings, the exclusive presence or dominance of the nonzoonotic *S. cruzi* in cattle tissues has been genetically confirmed in other studies conducted in Brazil (78 successfully molecularly characterized samples out of 78, [17]), Republic of Korea (31/31, [15]), Malaysia (17/17, [18]), Republic of China (215/216, [19]), Argentina (24/29, [1]), Hungary (23/36, [2]), Italy (285/384, [10]), Germany (134/257, [11]), and Iran (89/90, [22]), highlighting that this species is the most prevalent in cattle worldwide. However, caution should be taken in interpreting molecular survey results because the differences in the registered *Sarcocystis* spp. can be markedly influenced by the processed tissue matrices (e.g., myocardium, striated muscle, esophagus, diaphragm, and tongue). In this regard, the presence of the only species in the current survey is not surprising, considering that until now, the evidence of two other common *Sarcocystis* spp., namely, *S. hirsuta* and *S. hominis*, has not been molecularly confirmed in cattle myocardium samples [23]. Likewise, 17 out of 21 (81%) *S. cruzi* sequences have been isolated from cattle reared under the grazing system. Considering that domestic dogs (*Canis lupus familiaris*) are recognized as natural definitive hosts for *S. cruzi*, the intense usage of these carnivores in cattle management in grazing systems in our country results in a close relationship between the definitive and intermediate hosts. In addition, the free access of wild carnivores to pastures can favor the cattle contamination with the infective stages of the parasite. This could provide another explanation for the exclusive detection of this species in the current survey [18]. The complete life cycle of *S. hominis* and *S. hirsuta* in cattle requires the ingestion of sporocysts shed by primates and cats [23], a consequence of improper management of human sewage and environmental occurrence of unburied cat feces [18].

From the epidemiological point of view, based on the not-so-negligible prevalence (17.9%) found in cattle, the results depose for an environmental contamination with *S. cruzi* sporocysts, shed by infected carnivores. The sporocysts can resist and retain their infectivity in the environment for different external factors (e.g., freezing, high temperature, and several disinfectants), for a long period [23]. Therefore, the ubiquity of cattle exposure suggests that human beings also may ingest sporocysts of *S. cruzi*. Indeed, a *S. cruzi*-positive diarrheal sample was identified by microscopic and molecular examination in an AIDS-affected woman in Iran [24]. However, the clinical consequence of such exposure remains entirely unexplored. Likewise, the study provides useful insights for veterinarians regarding the importance of *S. cruzi* in bovine health populations in Romania. For instance, *S. cruzi* appears to be able to cause abortions that are clinically indistinguishable from those caused by *Neospora* and differentiation depends on immunohistochemistry or PCR [25]. In addition, *S. cruzi* is strongly suspected to be implicated in cases of fulminant death [2, 26], as well as in cases of eosinophilic myositis in cattle [7].

No correlation was found between the *Sarcocystis* infection positivity and the origin, age, breed, and gender of cattle, but the grazing farming system was positively associated (*p*=0.031) with the pathogen prevalence and can be considered a risk factor (OR = 3.6) in acquiring the infection. Thereby, a preliminary comparison of cattle husbandry indicates differential exposure to *S. cruzi*, a parasite excreted by dogs, resulting in a prevalence in grazed cattle, at least 10% greater. However, caution should be taken in interpreting the statistical results of the study because the limited number of processed samples can increase the margin of error and decrease the power of the analysis in discerning meaningful differences between the enrolled variables. According to our results, in other epidemiological surveys aimed to detect *Sarcocystis* infection in cattle, gender- and breed-related susceptibility to infection was not demonstrated (reviewed by [23]). Contrary to our findings, in a study conducted in Tunisia, Amairia et al. [27] reported a significantly higher (*p*=0.05) *S. cruzi* detection rate in animals aged between 2 and 8 years, compared to young animals. The same tendency has been observed by Yang et al. [19] in cattle older than 2 years in a survey conducted in central China. Moré et al. [14] have also reported higher values of sarcocysts per gram in the myocardium of Argentinean adult cattle, compared to young cattle. The longer and repetitive exposure of adult animals to infective sporocysts, compared to young ones, can support these findings.

The grazing farming system has a positive effect on the risk of *S. cruzi* infection. This observation can be sustained by the more intense perpetuation of the intermediate and definitive host relationship within the parasite's life cycle in this system, compared to the industrial one, based on the free access of cattle to infective forms of the parasites shed by definitive hosts (e.g., domestic and/or wild carnivores) [17]. Dogs excreting infective sporocysts contaminate the natural environment of grazing cattle, including feeders with silage and hay, the pasture, and water sources. Evidence of the parasite in cattle from the industrial system with restricted access for dogs may be related to the vehiculation of infective forms of the parasite in flies, which can transport the parasite from dog feces to cattle feed, as has been highlighted by Markus [28]. In addition, feeding alleys, stored grass, corn silage, and water contamination with sporocysts, due to management failures (e.g., accidental access of dogs kept in the neighborhood of farms), can also be considered a source of infection for cattle in the intensive farming system.

The structure of the resulting phylogenetic tree (Figure 2) revealed that the *S. cruzi* isolates, selected for the tree construction, clustered in several branches together with other cattle-specific and GenBank-deposited *Sarcocystis* species (*S. heydorni* and *S. hirsuta*) sequences from various parts of the world, a phenomenon which has been noted previously [29]. In the last decade, even if other more distinct sequences have subsequently been identified worldwide, the reported new data remain supportive of global dissemination of at least one invariant strain, perhaps with more diversity in Malaysia, in accordance with the findings published by Ng et al. [18]. It is also possible that more distinct sequences may eventually be attributed to other parasite species.

## 5. Conclusions

The results of this study highlighted that Romanian cattle destined for human consumption can harbor *S. cruzi* sarcocysts, with uniform distribution in the screened area, and the grazing farming system can be considered a risk factor in bovine sarcocystosis. The study provides useful information for veterinarians about the epidemiology of *Sarcocystis* infecting cattle in our country, although, for a possible public health hazard evaluation, a larger number of animals and other *Sarcocystis*-specific tissue matrices need to be screened. Likewise, investigations that highlight the presence of infections in humans are necessary for a better understanding of the zoonotic risk of sarcocystosis.

## Figures and Tables

**Table 1:** Distribution of *Sarcocystis cruzi* in the southwestern Romanian slaughtered cattle according to individual animal and epidemiological data.

Individual animal and epidemiological data	No. of sampled animals	No. of positive samples (%)	95% C.I. (lower-upper)	Odds ratios (OR) (95% CI)	*p* value
Counties					
Dolj	47	7 (14.9)	6.7–28.9	Reference	0.643
Timiș	30	5 (16.7)	6.3–35.5	0.9 (0.3–3.1)	
Gorj	40	9 (22.5)	11.4–38.9	1.5 (0.4–4.9)	

Age group (years)					
≤2	31	3 (9.7)	2.5–26.9	Reference	0.381
2 to 8	50	10 (20.0)	10.5–34.1	2.3 (0.6–9.3)	
>8	36	8 (22.2)	10.7–39.6	2.7 (0.6–11.1)	

Breed					
Holstein Friesian	32	5 (15.6)	5.9–33.6	Reference	0.875
Bălțată Românească	24	4 (16.7)	5.5–38.3	1.1 (0.3–4.5)	
Crossbreed	61	12 (19.7)	11.0–32.2	1.3 (0.4–4.2)	

Gender					
Female	102	18 (17.7)	11.1–26.7	Reference	0.825
Male	15	3 (20.0)	5.3–48.6	0.9 (0.2–3.4)	

Farming system					
Intensive	48	4 (8.3)	2.7–20.9	Reference	0.031
Grazing	69	17 (24.6)	15.4–36.7	3.6 (1.1–11.5)	

Total	117	21 (17.9)	11.7–26.3		

## Data Availability

The sequences (accession numbers MH223459 and MH223460) used to support the findings of this study have been deposited in the GenBank repository. The datasets generated and analyzed during the current study are included within the article.
